# Testing a Neuro-Evolutionary Theory of Social Bonds and Addiction: Methadone Associated With Lower Attachment Anxiety, Comfort With Closeness, and Proximity Maintenance

**DOI:** 10.3389/fpsyt.2019.00602

**Published:** 2019-09-06

**Authors:** Nuno Torres

**Affiliations:** Instituto Universitario de Ciencias Psicologicas Sociais e da Vida, William James Research Center, Lisbon, Portugal

**Keywords:** addiction, attachment, opioids, opiates, methadone, caregiving

## Abstract

Evidence from non-human mammals for the involvement of the endogenous opioid system in prosocial behavior is reasonably extensive and robust; however, studies in humans are lacking. This study tests the neuro-evolutionary hypothesis that exogenous opiates, including morphine, heroine, and methadone, decrease separation anxiety and proximity by hijacking the neuro-peptide endogenous opioid system modulating social bonds. Participants were 486 subjects, 43% male, with ages between 18 and 62 years (M = 26.4; SD = 9.4), divided in three naturalistic groups: 1: addicts in drug-free treatment; 2: addicts in methadone programs; 3: normative non-clinical controls.

**Instruments:** 1) Adult Attachment Scale (AAS) composed of three subscales: Anxiety about being rejected (α = 0.83), Comfort with Intimacy (α = 0.68), and Comfort Depending on Others (α = 0.70). 2) Caregiving Questionnaire composed of four subscales: Proximity Maintenance: (α = 0.83), Sensitivity: (α = 0.76), Controlling Caregiving (α = 0.77) and Compulsive Caregiving (α = 0.68).

**Results:** Multivariate Analysis of Co-variance (MANCOVA) models were computed; gender, age, and education were included in the models. Methadone patients and drug-free treatment addicts were equivalent and reported significantly lower Comfort Depending on Others, Comfort with Intimacy, and Caregiving Proximity. However, methadone users reported significantly lower Anxiety about being rejected than drug-free addicts and were equivalent to non-clinical controls. In addition, correlations between the methadone intake dose and the questionnaires’ scales showed that dose was significantly and negatively correlated with Comfort with Closeness (rs = −0.36; p < 0.01) and with Caregiving Proximity (rs = −0.28; p < 0.05).

## Introduction

Humans need intimate relationships of great depths of emotional, psychological and physical intensity for survival, and emotional well-being across the life cycle. Young children exhibit intense crying when helpless, lonely, or lost, alerting caretakers to attend to their needs. Adolescents and adults look for support, emotional and sexual bonding in social interactions and relationships, without which they feel empty and alienated. Social mammals need these affiliative interactions in order to get relief from negative emotions but also to get pleasure and joy (1).

It is now widely consensual that being able to form positive socio-emotional bonds has implications for physical and mental health as well as for greater social competence. Dysfunctional relationships, social rejection, and withdrawal are associated with a wide range of psychopathologies including drug abuse, anxiety, and depression (2, 3).

Research evidence in the last decades showed that the need for social bonding is neurologically hard wired in socially dependent animals, including humans (4, 5). Specifically, there has been vast research on the neurochemical bases of parental and romantic social bonds focused on the neuropeptides oxytocin, vasopressin, dopamine, and serotonin (6, 7).

Additionally, and based upon the homologies between opioid drug addiction and romantic bonding (8, 9), some authors have pointed out the endogenous opioids as another group of neurochemical mechanism motivating parental and relationship behavior in humans. These homologies are quite remarkable: they are both characterized by an initial strong attraction (i.e., the euphoria stage), which then decreases with exposure (i.e. the tolerance stage). After the emergence of tolerance, the system adapts to a new “set point” whereby absence of the partner/substance leads to negative affect and distress symptoms that are similar for opiate withdrawal and for social loss (10, 11).

Several studies with rodents using self-administration showed that the lack of social bonding due to isolation enhanced the consumption of opiates (12, 13). Also, opiates and opioids have shown to be effective in reducing separation distress, in puppies, young guinea pigs, and chicks, while opiate antagonists increase vocalizations induced by separation (14).

Additionally, it is now established by the concept of “social pain” that social bonding/rejection and physical pain share similar neuronal pathways (15). This area of research suggested that responses to positive and negative events on social interactions are regulated by endogenous opioid peptides and the μ-opioid receptor, which also alleviates physical pain (16). The μ-opioid receptor (MOR) system has also been shown to interact with oxytocin and dopamine in social bonding and social reward (17, 18). This is likely explained by the adaptive value of the social attachment system, which keeps young close to parents, and may have evolved to enhance biological fitness in social animals (19).

Starting from this brain opioid theory of social attachment, Panksepp et al. (10,,) suggested that opiate addiction (morphine, heroin, etc.) could be neurologically motivated in part by the capacity of these drugs to reduce the pain and the lack of joy of inadequate social bonding and attachments. On the other hand, opiates’ consumption reduces the drive for social interactions in animals, including humans, while small doses increase feelings of confidence and social dominance (10, 11). It is also known that the repeated use of opiates in its turn induces alterations in neurotransmitter and neuropeptide systems in brain circuits that regulate mood and affect (21).

The attachment theory (22, 23) has been applied widely as a theoretical framework for understanding how close interpersonal bonds can shape both normal and abnormal development. According to this tradition, humans are innately equipped with behavioral systems for social attachment and caregiving, since being emotionally bonded to parents, friends, romantic partners, and providing care for dependent individuals enhanced genetic success or inclusive fitness (24).

The attachment theory tradition has provided several measurement methods such as the Adult Attachment Interview (AAI) and a series of self-report questionnaires, such as the Adult Attachment Scale (AAS) (25) and the Caregiving Questionnaire (26), to access individual differences in psycho-social close relationships.

Translating these notions to human addiction studies, in previous works, we found that addicts vs controls recalled a significantly greater number of traumatic events in childhood and adolescence (such as parental death, child abuse, and early separation), which are known to severely disrupt the attachment system (27). They also had higher scores on attachment Anxiety and Avoidance of close relationships, and additionally, these scores were significantly correlated with the number of traumatic family events in childhood and adolescence (28).

Recently, a meta-analysis found both cross sectional and prospective significant correlations between attachment and (later) substance use, albeit both of small magnitude; these results indicate that lower attachment security is concurrent to and temporally preceded increases in substance use (29). Additionally, the study found no evidence of a moderation effect of the type of attachment measure—e.g., AAI, AAS—on the correlation between attachment and substance use.

Although there is today a vast amount of studies showing a robust association between subjects with a diagnosis of drug addiction and severe problems in close relationships, there is not to our knowledge a comparative study between addicts in opiate abstinence vs addicts consuming the opiate methadone, vs non-addicted controls. There is also a lack of studies focusing specifically on individual differences in profiles of the caregiving system (25).

## Objectives

In the present cross-sectional comparative study, we aimed to test the effect of the opiate agonist methadone use and dosage on measures of two behavioral systems hypothesized by Bowlby (23) to regulate close social bonds (the Attachment system and the Caregiving system).

## Hypotheses

1. Opiate-addicted subjects have close relationship profiles characterized by higher avoidance of close proximity in social bonds and higher attachment-related anxiety than non-clinical controls.
2. Methadone intake and dosage are associated with lower self-reported attachment-related anxiety and higher avoidance of proximity maintenance in close relationships

## Participants

A total of 486 subjects participated in the study; 43% were male, their age ranging from 18 to 62 years (M = 26.4; SD = 9.4). Participants were members of three naturalistic groups: Group 1: addicts in drug-free (DF) treatment therapeutic communities (n = 56); Group 2: addicts in MMT-Methadone maintenance treatment (n = 88); Group 3: normative non-clinical controls (n = 342). The participants in group 1 were residents of three therapeutic communities (TC) in Portugal that adhere strictly to total abstinence and drug-free policies, with few exceptions for a minority of patients that could not withdraw methadone (the patients taking methadone in the TC were excluded from the study statistics). Participants in group 2 were addicts in outpatient treatment and outreach programs in Lisbon, taking daily doses of methadone under medical supervision. The methadone dose ranged from 5 to 215 mg (M = 65.8 mg; SD = 38.6 mg); these dose values are of similar range and average to other studies [e.g., Ref. (30)]. Participants in group 3 were Psychology university students in Lisbon.

Due to the lack of previous studies comparing attachment variables on methadone users, abstinent substance abusers, and non-clinical subjects, it was impossible to rely on a reasonably expected effect size. This fact prevented us from doing an a priori power analysis to estimate the minimum N of the sample. Hence, we used rules of thumb from the literature according to which, in a variety of settings, the minimum number of subjects per variable lies in the range of 15 to 20 (31, 32). The non-clinical group subjects were part of a larger study on attachment and caregiving in university students; for that reason, the number of subjects was substantially higher.

Groups 1 and 2 were not significantly different in gender, educational level, age started abusing drugs, percentage of father, mother, and siblings with substance abuse problems, and total number of relatives with substance abuse problems. Group 1 was slightly older than group 2, and group 3 was younger, had more years of education, and contained more females than the other two groups (all differences p < 0.05). **Table 1** shows the demographic characteristics of the participants. **Supplementary Table 1** shows additional characteristics of the addiction subjects. The two groups of addicts were equivalent in all variables except “Father with addiction” and Methadone intake.

**Table 1:** Demographic characteristics of participants.

	Group		
	Addicts in DF Treatment	Addicts in Metadone MT	Non-clinical
Sex (% male)	65^a^	69^b^	32^c^
Age	34,5^a^	38,1^a^	22,1^b^
Education (years of)	8,9^a^	8,5^a^	13,1^b^

*Values with different letters are significantly different at p < 0.05.*

## Ethical Approval

All participants provided written informed consent to participate in the study. All procedures were approved by the Research Ethics Committee of the university (ISPA—Instituto Universitário, Lisbon, Portugal) and in accord with the ethical principles of psychologists and code of conduct of the American Psychological Association.

## Instruments

Subjects completed a battery of two self-report questionnaires. The order of the questionnaires was randomly counterbalanced:

1. Adult Attachment Scale (AAS) (26) consists of 18 items scored on a 5-point Likert-type scale. We used the Portuguese version, adapted by Canavarro, Dias and Lima (33). The questionnaire contains three subscales, each composed of six items. The three subscales are CLOSE, DEPEND, and ANXIETY. The CLOSE scale measures the extent to which a person is comfortable with closeness and intimacy (e.g., “I do not worry about someone getting too close to me”). The DEPEND scale measures the extent to which a person feels he/she can depend on others to be available when needed (e.g., “I know that people will be there when I need them.”). The ANXIETY subscale measures the extent to which a person is worried about being abandoned or unloved (e.g., “I do worry about being abandoned”.). The psychometric consistency of the scales in the present study was as follows: ANXIETY about being rejected or unloved (α = 0.83), CLOSE—Comfort with Closeness and Intimacy (α = 0.65) and DEPEND—Comfort Depending on others (α = .70).
2. Caregiving Questionnaire (1) consists of 32 items scored on a 6-point Likert scale, assessing caregiving behaviors in romantic and marital relationships. We used the Portuguese version, adapted by Torres and Oliveira (34). It is composed of four subscales: The Proximity maintenance (or Proximity vs Distance) subscale assesses the degree to which subjects make themselves available to their partner when comfort is needed (e.g., “When my partner seems to want or need a hug, I’m glad to provide it”). The Sensitivity subscale assesses the degree to which subjects recognize when their partner needs support (e.g., “I can always tell when my partner needs comforting, even when s/he doesn’t ask for it”). The Controlling subscale measures the degree to which subjects exert control to help their partners solve problems (e.g., “I tend to be too domineering when trying to help my partner”). Finally, the Compulsive subscale measures the extent to which subjects get over-involved in their partners problems (e.g., “I sometimes create problems by taking on my partner’s troubles as if they were my own”). The psychometric consistency of the scales in the present study was as follows: Proximity Maintenance: (α = 0.83), Sensitivity: (α = 0.76), Controlling Caregiving (α = 0.77), and Compulsive Caregiving (α = 0.68).

The addicted subjects further completed the section D of Portuguese ASI-6 (Addiction Severity Index, Version 6) by a clinical psychologist member of the research team in order to check if all them had heroin as a drug of addiction, which was the case.

## Data Analysis Plan

All statistics were performed using the IBM SPSS Statistics package Version 21.0. Preliminary inspection of the data showed that the AAS and Caregiving questionnaires’ scales were normally distributed, while the methadone intake variable significantly differed from the normal distribution.

First, we performed Pearson correlations between all questionnaire scales in study, in order to test for theoretically congruent associations between attachment and caregiving constructs, and to detect potential multicollinearity (which was not present: all correlation coefficients were below .50). Second, in order to test for differences between the three groups, two Multivariate Analysis of Co-variance (MANCOVA) models were computed, one with the AAS and one with the Caregiving scales as dependent variables; the three groups of subjects were included as the independent variable. The variables Sex, Age, and Education were also included in the models as covariates to statistically control for the demographic differences between the three groups.

Finally, we tested the association between methadone dosage in milligrams and all the questionnaire scales using Spearman non-parametric correlations since methadone dosage had a non-normal distribution.

## Results

The intercorrelation matrix, presented in **Supplementary Table 2**, shows theoretically congruent significant correlations between the constructs of attachment and caregiving.

Two MANCOVA models were computed, one with the AAS scales as dependent variables and the other with the Caregiving scales as dependent variables. In both models, the three groups of subjects were the levels of the independent variable, and the variables Sex, Age, and Education were included in both models. All MANCOVA assumptions were tested and met by the data except the equality of variances, which were significantly different in the AAS Capacity to be Close scale (F = 3.45; p = 0.004) and the Caregiving Controlling scale (F = 2.90; p = 0.013). For this reason, we performed the MANCOVAs using a bootstrap method (35) with the number of samples = 1,000, available in the SPSS package. In both models, multivariate tests showed significant effects of the group variable only, no significant main effects of the demographic variables, and no interaction effects between the variables in the model. **Table 2** shows the mean differences for each group on all the questionnaires’ scales, the value of F statistic, and contrasts for both models.

**Table 2:** MANCOVA models’ results and differences between groups on Attachment and Caregiving variables.

	Addicts in DF Treatment	Addicts in Metadone MT	Non-clinical Subjects	F_2.451_	*p*	Partial Eta Squared
**Model 1: AAS**						
Anxiety about rejection	3.42^a^ (0.83)	2.39^b^ (0.76)	2.44^b^ (0.78)	29.91	0.000	0.116
Capacity to be Close	3.03^a^ (0.51)	2.88^a^ (0.68)	3.68^b^ (0.49)	18.77	0.000	0.076
Comfort Depending on others	2.82^a^ (0.58)	2.72^a^ (0.66)	3.19^b^ (0.57)	7.68	0.001	0.033
**Model 2: CAREGIVING**						
4. Proximity maintenance	3.91^a^ (0.65)	3.95^a^ (0.99)	4.81^b^ (0.88)	6.68	0.001	0.029
5. Sensitivity	3,65^a^ (0.65)	3.86^a^ (0.98)	4.32^b^ (0.73)	5.47	0.005	0.024
6. Controlling Caregiving	3.97^a^ (0.66)	3.61^a^ (1.01)	2.88^b^ (0.82)	21.83	0.000	0.090
7. Compulsive Caregiving	3.65^a^ (0.75)	3.41^a^ (0.97)	3.19^b^ (0.76)	3.53	0.030	0.016

Values with different superscripted letters are *post hoc* significantly different at p < 0.05.

Standard Deviations in parenthesis below means.

As can be seen in **Table 2**, the Methadone intakers and Drug-free treatment addicts were statistically equivalent on all questionnaire scales, except for the AAS scale “Anxiety about being rejected or unloved”: in this scale, the Methadone MT subjects had significantly lower scores than drug-free addicts and were equivalent to non-clinical controls.

Finally, we tested the association between methadone dosage in milligrams and all the questionnaire scales using Spearman non-parametric correlations.

**Figure 1:**
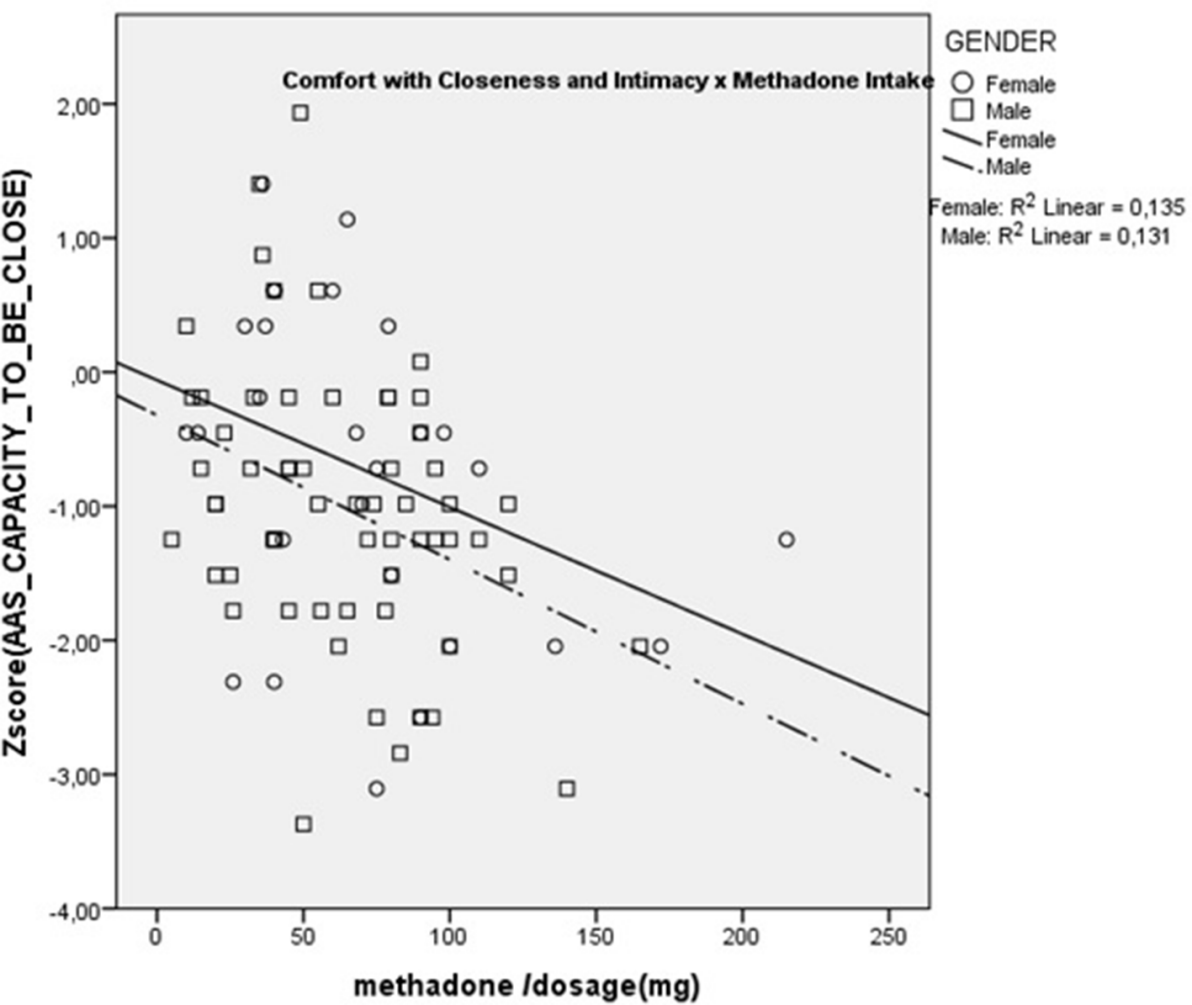
Scatterplot of Correlation between methadone dose and Capacity to be Close.

**Figure 2:**
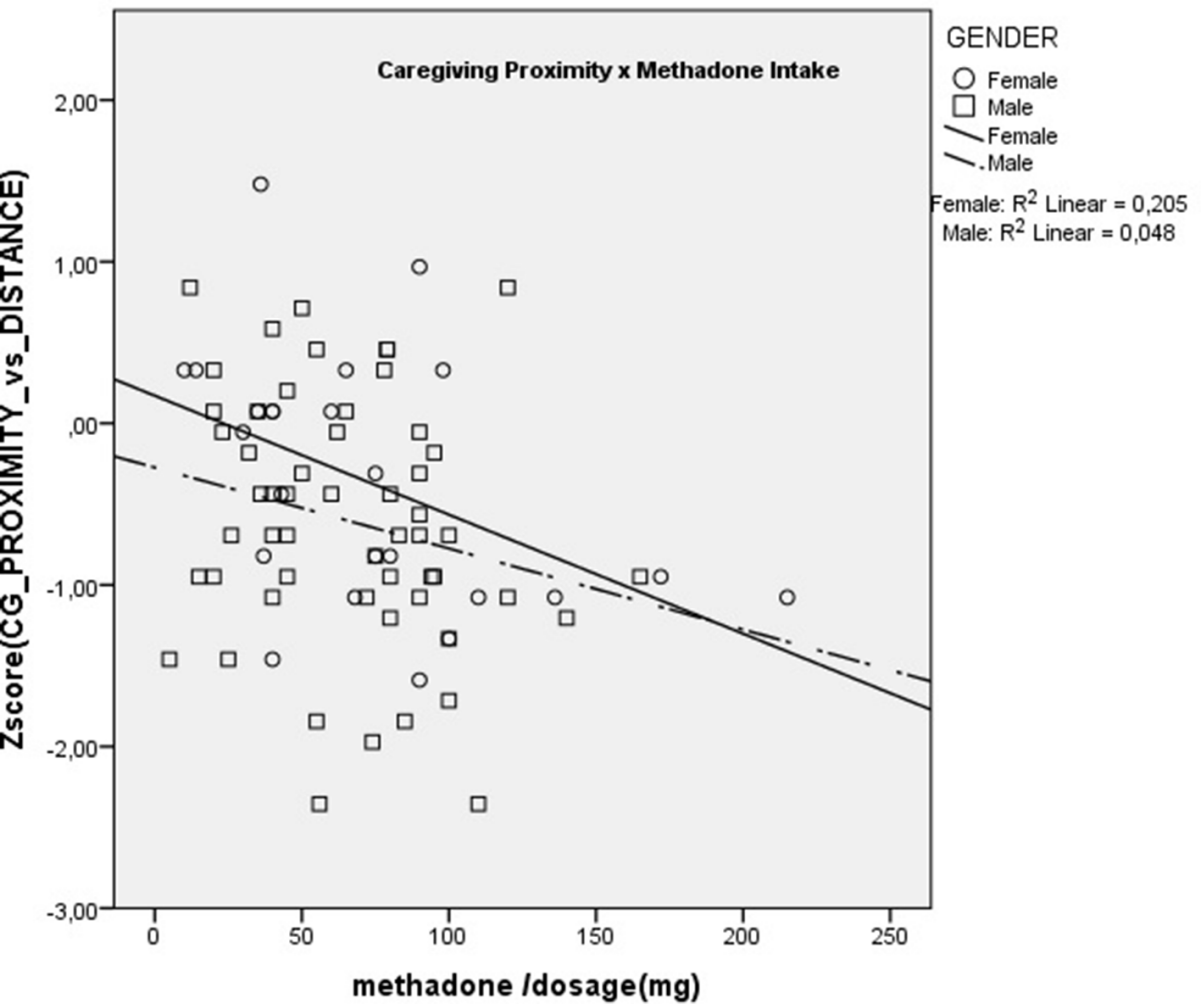
Scatterplot of Correlation between methadone dose and Caregiving Proximity.

Results showed that dose was significantly and negatively correlated with AAS Comfort with Closeness (r_s_ = –0.36; p < 0.01) and with Caregiving Proximity (r_s_ = –0.28; p < 0.05). There were no significant correlations with any of the other scales. These are presented in **Figures 1** and **2**. These results show that increasing methadone doses were associated with diminished capacity for, and diminished comfort with, emotional and physical closeness with partners.

## Discussion

The results of our study show support for the neuro-evolutionary theory of social bonds and addiction [also known as Brain Opioid Theory of Social Attachment (BOTSA)] (4), according to which exogenous opiates decrease separation anxiety and proximity maintenance in humans, as in animal models, by hijacking the neuro-peptide endogenous opioid system modulating social bonds (12).

In our sample, both groups of drug addicts—more than 95% reported the opiate heroin as their main addiction—showed lower levels of adaptive profiles of attachment and caregiving compared with non-clinical controls. This result is congruent with more than 20 cross-sectional studies, which have reported that as attachment security decreases, substance use increases (29). Similarly to these previous studies, in the present study, the estimated effect sizes were in the small-to-medium range (the Partial Eta Squared ranged from 0.016 to 0.116).

We had the possibility of comparing two groups of subjects with equivalent histories of opiate addictions: 1—addicts currently in abstinence of opiates and 2—methadone intakers. Methadone is a synthetic opioid that acts on the same opioid receptors as morphine and heroin. It is commonly used to treat opiate addictions, especially addiction to heroin, and has been considered by some as the “gold standard” for treating opiate addiction (36). The abstinent drug addicts were inpatients at therapeutic communities’ residential treatment with strict abstinence rules for all drugs including alcohol, undergoing regular urine analyses to detect drugs. For this reason, we can have a high degree of confidence that they were actually abstinent of opioids. In this way, we had the opportunity to compare in a quasi-experimental way, the effect of an opiate drug on self-reported psychological states related to intimate social bonds and attachment.

Results showed that the methadone users reported significantly less feelings of attachment anxiety, i.e., anxiety about being abandoned or unloved, than their abstinent counterparts. Furthermore, this association had the stonger effect size of all questionnaire scales (Partial Eta Squared = 0.116), which represents a medium size effect. This result is congruent with experimental work on animal models, which showed that opiate agonists decreased observable signs of anxiety due to separation and isolation (14, 37). In a range of mammals, including rats, mice, chicks, sheep, guinea pigs, dogs, non-human primates, and humans, separation from the mother leads the young to emit distress vocalizations. There is considerable evidence from a range of species that administration of morphine reduces these vocalizations, while the opioid antagonist naloxone increases them (4). The fact that in our study the methadone intakers were statistically equivalent to the abstinent addicts, except for the lower score of AAS separation anxiety, gives us some grounds to suggest a possible homology with the opioid-mediated separation distress paradigm in animal models.

Additionally, we were able to correlate methadone dosage with the attachment and caregiving scales, within the methadone intakers group. Results showed that higher methadone dosage was associated with lower levels of Caregiving Proximity and Comfort with Closeness. The Caregiving Proximity maintenance subscale is a measure of the degree to which subjects make themselves available to their partners when comfort is needed and, hence, is an important part of parental-like behaviors. The negative correlation with methadone dosage is congruent with previous animal studies showing that morphine significantly impairs parental behavior such as retrieving, grouping, licking, and nursing the young, while naloxone, an opiate antagonist, restores it (38). Opiates, in particular mu-receptor ligands, disrupt maternal behavior in a very selective, naloxone-reversible fashion (39). Additionally, the negative correlation of Comfort with closeness suggests that methadone might decrease the rewarding aspect of physical contact characteristic of parental and affiliative behaviors, a phenomenon that was previously suggested for other opiates (40). These results with the notion that patients maintained on opioids relate autistically (e.g., “with coldness in human interactions and gaze avoidance) which are reversed by detoxification from opioids” (41).

The absence of significant correlation between higher methadone doses and lower attachment anxiety might at first sight be seen as counter-intuitive and contradicting the other results. However the mean value of AAS-Anxiety in the MMT group was below the total sample mean (as the non-clinical subjects), which reduces statistical variance and may contribute to a non-significant correlation. On the other hand, it is also likely that methadone can reduce attachment-anxiety/separation distress at low doses, and hence, increasing the dosage does not have a proportional effect due to a ceiling effect. Indeed, it has been reported in psychiatric patients that “opiates at low doses can powerfully counteract feelings of social loss and despair” (p. 645) (12), in rodents “low doses of morphine inhibit separation distress of infants” (4), as well as low doses of opiates (down to 0.5 mg/kg) can reduce motivation to social contact (40). These results converge with previous literature suggesting that provision of exogenous opioids, such as methadone, may have significant long-term consequences of degrading the endogenous opioid system such as avoidance of social interactions “that are not currently accounted for in medical practice” (41).

It is worth noting that as in previous studies, the obtained effect sizes were in the small-to-medium range. This fact is congruent with the notion that opiate drugs’ abuse/addiction is a multifactorial phenomenona with a great number of both genetical and environmental determinants. For this reason, effect sizes for specific biopsychosocial risk factors may often emerge as small in magnitude (29, 42). Another possible reason for the small effect sizes is that there might be moderators of the attachment–drug abuse association at several levels of analysis (biological, genetic, psycho-social, geographical, macro-social). However, in this study, we did not find significant interactions between gender, education or age and drug abuse status. A meta-analysis by Fairbairn et al. (29) tested a large number of potential moderators (age, gender, racial composition, geographic region, substance use pattern, attachment figure, attachment measures, and others) and found only age to be a moderator in prospective studies but not in cross-sectional studies, like the present one. We need more multilevel studies that can address the interactions between genetic, epigenetic, neurobiological factors, and experiences of psychosocial and relationship adversity at several stages of development, to reframe our understanding of how attachment/close relationship variables are moderated by other phenomena in the development, severity, and maintenance of addiction (43).

The present study has a number of limitations. First, the ones which are typical of cross-sectional correlational and quasi-experimental designs: it is not possible to determine or infer directional causality from the data. Additionally, the use of self-report questionnaires to measure attachment-theory constructs has its own limitations and has drawn criticism: on the one hand, some of the psychological processes are supposed to take place implicitly or unconsciously and, hence, cannot be measured by explicit self-report measures; on the other hand, previous research showed that the correlations between implicit measures of attachment such as in-depth interviews and self-report questionnaires are typically low (44). Furthermore, it is possible that respondents manipulate some of their answers, either in a conscious or unconscious way. However, the meta-analysis by Fairbairn et al. (29) did not found any significant influence of the different attachment measures (i.e., implicit measures such as the AAI and explicit measures such as the AAS questionnaire) on the results, which gives us some reassurance that the present results may be robust.

## Data Availability

The datasets generated for this study are available on request to the corresponding author.

## Ethics Statement

The studies involving human participants were reviewed and approved by ISPA-IU: Comité de Ética do Centro de Investigação. The patients/participants provided their written informed consent to participate in this study.

## Author Contributions

The author confirms being the sole contributor of this work and has approved it for publication.

## Funding

This research was funded by grants from the Foundation for Science and Technology of Portugal: UID/PSI/04810/2019.

## Conflict of Interest Statement

The author declares that the research was conducted in the absence of any commercial or financial relationships that could be construed as a potential conflict of interest.

## References

[1] Loseth GE Ellingsen DM Leknes S State-dependent μ-opioid modulation of social motivation. Front Behav Neurosci (2014) 8:430. 10.3389/fnbeh.2014.00430 25565999 PMC4264475

[2] Machin AJ Dunbar R The brain opioid theory of social attachment: a review of the evidence. Behaviour (2011) 148:985–1025. 10.1163/000579511X596624

[3] Hsu DT Sanford BJ Meyers KK Love TM Hazlett KE Wang H Response of the μ-opioid system to social rejection and acceptance. Mol Psychiatr (2013) 18(11):1211–17. 10.1038/mp.2013.96 PMC3814222 23958960

[4] Panksepp J Herman B Conner R Bishop P Scott JP The biology of social attachments: opiates alleviate separation distress. Biol Psychiatr (1978) 13:607–18. 83167

[5] Panksepp J Why does separation distress hurt? Comment on MacDonald and Leary (2005). Psychol Bull (2005) 131(2):224–30. 10.1037/0033-2909.131.2.224 15740418

[6] Carter CS DeVries AC Getz LL Physiological substrates of mammalian monogamy: the prairie vole model. Neurosci Biobehav Rev (1995) 16:131–44. 10.1016/0149-7634(94)00070-h 7630584

[7] Insel TR Young LJ Neuropeptides and the evolution of social behavior. Curr Opin Neurobiol (2000) 10:784–9. 10.1016/S0959-4388(00)00146-X 11240290

[8] Liebowitz MR Chemistry of love. Boston, MA: Little Brown (1983).

[9] Panksepp J Affective neuroscience. New York, NY: Oxford University Press (1999).

[10] Panksepp J Knutson B Burgdorf J The role of brain emotional systems in addictions: a neuro-evolutionary perspective and new “self-report” animal model. Addiction (2002) 97:459–69. 10.1046/j.1360-0443.2002.00025.x 11964061

[11] Panksepp L Nocjar C Burgdorf J Panksepp JB Huber R The role of emotional systems in addiction: a neuroethological perspective. Nebr Symp Motiv (2004) 50:85–126. 15160639

[12] Alexander BK Beyerstein BL Hadaway PF Coambs RB Effect of early and later colony housing on oral ingestion of morphine in rats. Pharmacol Biochem Behav (1981) 15:571. 10.1016/0091-3057(81)90211-2 7291261

[13] Bozarth MA Murray A Wise RA Influence of housing conditions on the acquisition of intravenous heroin and cocaine self-administration in rats. Pharmacol Biochem Behav (1989) 33:903–7. 10.1016/0091-3057(89)90490-5 2616610

[14] Panksepp J Herman BH Vilberg T Bishop P DeEskinazi FG Endogenous opioids and social behavior. Neurosci Biobehav Rev (1978) 4:473–87. 10.1016/0149-7634(80)90036-6 6258111

[15] Eisenberger NI Lieberman MD Williams KD Does rejection hurt? An FMRI study of social exclusion. Science (2003) 302:290–292. 10.1126/science.1089134 14551436

[16] Panksepp J Herman BH Vilberg T Bishop P DeEskinazi FG Endogenous opioids and social behavior. Neurosci Biobehav Rev (1980) 4(4):473–87. 10.1016/0149-7634(80)90036-6 6258111

[17] Depue RA Morrone-Strupinsky JV A neurobehavioral model of affiliative bonding: implications for conceptualizing a human trait of affiliation. Behav Brain Sci (2005) 28:313–50. 10.1017/S0140525X05000063 16209725

[18] Tops M Koole SL Ijzerman H Buisman-Pijlman FTA Why social attachment and oxytocin protect against addiction and stress: insights from the dynamics between ventral and dorsal corticostriatal systems. Pharmacol Biochem Behav (2014) 119:39–48. 10.1016/j.pbb.2013.07.015 23916423

[19] Nelson EE Panksepp J Brain substrates of infant–mother attachment: contributions of opioids, oxytocin and norepinephrine. Neurosci Biobehav Rev (1998) 22:437–52. 10.1016/S0149-7634(97)00052-3 9579331

[20] Panksepp J Commentary on “Understanding Addictive Vulnerability”. Neuro-Psychoanal (2003) 5(1):21–9. 10.1080/15294145.2003.10773404

[21] De Vries TJ Shippenberg TS Neural systems underlying opiate addiction. J. Neurosci. (2002) 22(9):3321–5. 10.1523/JNEUROSCI.22-09-03321.2002 PMC6758389 11978806

[22] Bowlby J Attachment and loss: attachment. New York, NY: Basic Books (1969).

[23] Ainsworth MDS Blehar MC Waters E Wall S Patterns of attachment: Assessed in the strange situation and at home. Hillsdale, NJ: Erlbaum (1978).

[24] Mikulincer M Shaver PR Gillath O Nitzberg RA Attachment, caregiving, and altruism: boosting attachment security increases compassion and helping. J Pers Soc Psychol (2005) 89(5):817–39. 10.1037/0022-3514.89.5.817 16351370

[25] Collins N Read SJ Adult attachment, working models, and relationship quality in dating couples. J Pers Soc Psychol (1990) 58(4):644–63. 10.1037//0022-3514.58.4.644 14570079

[26] Kunce LJ Shaver PR An attachment-theoretical approach to caregiving in romantic relationships. In: Perlman D Bartholomew K, editors. Attachment processes in adulthood: Vol.5 Advances in personal relationships. vol. 5 Jessica Kingsley Publishers, Ltd. (1994).

[27] Torres N Disorders of Emotional Containment and their Somatic Correlates. The Protomental Nature of Addictions, Self-harm and Noncommunicable Diseases. PhD Thesis. Colchester, UK: Centre for Psychoanalytic Studies: University of Essex (2008).

[28] Torres N Sanches M Neto D Experiências traumáticas e estilos de vinculação adulta a parceiros de intimidade em toxicodependentes e estudantes. Toxicodependências (2004) 10(3):57–70.

[29] Fairbairn CE Briley DA Kang D Fraley RC Hankin BL Ariss T A meta-analysis of longitudinal associations between substance use and interpersonal attachment security. Psychol Bull (2018) 144(5):532–55. 10.1037/bul0000141 PMC5912983 29494194

[30] Trafton JA Minkel J Humphreys K Determining effective methadone doses for individual opioid-dependent patients. PLoS Med (2006) 3(3):e80. 10.1371/journal.pmed.0030080 16448216 PMC1360079

[31] Green S How many subjects does it take to do a regression analysis. Multivariate Behav Res (1991) 26(3):499–510. 10.1207/s15327906mbr2603_7 26776715

[32] Harrell F.E. Jr. Regression modeling strategies. New York, NY: Springer-Verlag (2001). 10.1007/978-1-4757-3462-1

[33] Canavarro MC Dias P Lima V A Avaliação da Vinculação do Adulto: uma revisão crítica a propósito da aplicação da Adult Attachment Scale-R (AAS-R) na população Portuguesa. Psicologia (2006) 20(1):155–86. 10.17575/rpsicol.v20i1.381

[34] Torres N Oliveira D Vinculação e Sistema de Prestação de cuidados em dependentes de substâncias em tratamento. Adaptação Portuguesa do Questionário de Prestação de Cuidados. Toxicodependências (2010) 16(2):3–14.

[35] Zientek L Thompson B Applying the bootstrap to the multivariate case: bootstrap component/factor analysis. Behav. Res. Methods (2007) 39:318–25. 10.3758/BF03193163 17695360

[36] National Institutes of Health (NIH) NIH Consensus Statement, 17–19 November. Effective Medical Treatment of Opiate Addiction Vol. 15 Bethesda, MD: NIH (1997) p. 1–38.

[37] Herman BH Panksepp J Effects of morphine and naloxone on separation distress and approach attachment: evidence for opiate mediation of social affect. Pharmacol Biochem Behav (1978) 9:213–20. 10.1016/0091-3057(78)90167-3 568801

[38] Stafisso-Sandoz G Polley D Holt E Lambert KG Kinsley CH Opiate disruption of maternal behavior: morphine reduces, and naloxone restores, cfos activity in the medial preoptic area of lactating rats. Brain Res Bull (1998) 45(3):307–13. 10.1016/S0361-9230(97)00375-4 9510424

[39] Mann PE Kinsley CH Bridges RS Opioid receptor subtype involvement in maternal behavior in lactating rats. Neuroendocrinology (1991) 53:487–92. 10.1159/000125762 1651460

[40] Panksepp J Nelson E Bekkedal M Brain systems for the mediation of social separation-distress and social-reward: evolutionary antecedents and neuropeptide intermediaries. Ann N Y Acad Sci (1997) 807:78–100. 10.1111/j.1749-6632.1997.tb51914.x 9071345

[41] Johnson B Ulberg S Shivale S Donaldson J Milczarski B Faraone SV Fibromyalgia, autism, and opioid addiction as natural and induced disorders of the endogenous opioid hormonal system. Discov Med (2014) 18(99):209–20. 25336035

[42] Hawkins JD Catalano RF Miller JY Risk and protective factors for alcohol and other drug problems in adolescence and early adulthood: implications for substance abuse prevention. Psychol Bull (1992) 112(1):64–105. 10.1037//0033-2909.112.1.64 1529040

[43] McCrory EJ Mayes L Understanding addiction as a developmental disorder: an argument for a developmentally informed multilevel approach. Curr Addict Rep (2015) 2(4):326–30. 10.1007/s40429-015-0079-2 PMC4628052 26550551

[44] Shaver PR Mikulincer M What do self-report attachment measures assess? In: Rholes WS Simpson JA, editors. Adult attachment: Theory, research, and clinical implications. Guilford Publications (2004). p. 17–54.

