# Supplementary material for: Testing a Neuro-Evolutionary Theory of Social Bonds and Addiction: Methadone Associated With Lower Attachment Anxiety, Comfort With Closeness, and Proximity Maintenance
Source: Front Psychiatry. 2019 Sep 6;10:602. doi: 10.3389/fpsyt.2019.00602 (PMC6743610; doi:10.3389/fpsyt.2019.00602)
Supplement: Supplementary file 1 [file Table_1.docx]

| Table S1. *Additional characteristics of addicted participants* | | | |
| --- | --- | --- | --- |
|  | **Addicts in DF Treatment** | **Addicts in Methadone MT** |  |
| **Age started abusing drugs** | 17 ^a^ | 18 ^a^ |  |
| **Heroin is main addiction (%)** | 95,2 ^a^ | 97,0 ^a^ |  |
| **Cocain is main addiction (%)^1^** | 4,8 ^a^ | 3,0 ^a^ |  |
| **Father had addiction problems (%)** | 32,3 ^a^ | 12,0 ^b^ |  |
| **Mother had addiction problems (%)** | 9,0 ^a^ | 14,1 ^a^ |  |
| **Sibling/s had addiction problems (%)** | 32,3 ^a^ | 43,1 ^a^ |  |
| **Has taken methadone (%)** | 39,1 ^a^ | 100,0 ^b^ |  |
| *Notes:*  *values with different letters are significantly diferent at p<.05*  ***^1^****- All “cocaine is main addiction” subjects mentioned also having Heroin as an addiction* | | |  |
