# Supplementary material for: Testing a Neuro-Evolutionary Theory of Social Bonds and Addiction: Methadone Associated With Lower Attachment Anxiety, Comfort With Closeness, and Proximity Maintenance
Source: Front Psychiatry. 2019 Sep 6;10:602. doi: 10.3389/fpsyt.2019.00602 (PMC6743610; doi:10.3389/fpsyt.2019.00602)
Supplement: Supplementary file 2 [file Table_2.docx]

| Table S2. *Pearson correlations between Attachment and Caregiving scales and descriptive statistics* | | | | | | | |
| --- | --- | --- | --- | --- | --- | --- | --- |
|  | **AAS Attachment Scales** | | | **Caregiving** **Scales** | | | |
| ***AAS*** | 1. | 2. | 3. | 4. | 5. | 6. | 7. |
| **1. Anxiety about rejection** |  | -0.18** | -0.21** | -0.12** | -0,15** | 0.32** | 0.27** |
| **2. Capacity to be Close** |  |  | 0.46** | 0.44** | 0.33** | -0.40** | -0.09 |
| **3. Comfort Depending others** |  |  |  | 0.40** | 0,30** | -0,28** | 0,03 |
| ***CAREGIVING*** |  |  |  |  |  |  |  |
| **4. Proximity maintenance** |  |  |  |  | 0,63** | -0,32** | 0,20** |
| **5. Sensitivity** |  |  |  |  |  | -0,23** | 0,17** |
| **6. Controlling Caregiving** |  |  |  |  |  |  | 0,44** |
| **7. Compulsive Caregiving** |  |  |  |  |  |  |  |
| **Descriptives** |  |  |  |  |  |  |  |
| Min-max | 1.0-4.7 | 1.3-4.7 | 1.0-4.2 | 1.0-6.0 | 1.0-6.0 | 1.0-5.6 | 1.0-5.4 |
| Mean (SD) | 2.6 (0.9) | 2.9 (0.7) | 2.8 (0.7) | 3.9 (0.9) | 3.8 (0.9) | 3.7 (0.9) | 3.5 (0.9) |
| *Note: ** p< 0.01 (2-tailed).* | | | | | | | |
